# Supplementary material for: Ultra-low loss quantum photonic circuits integrated with single quantum emitters
Source: Nat Commun. 2022 Dec 12;13:7693. doi: 10.1038/s41467-022-35332-z (PMC9744872; doi:10.1038/s41467-022-35332-z)
Supplement: Supplementary file 1 — Supplementary Information [file 41467_2022_35332_MOESM1_ESM.pdf]

Supplementary Information to "Ultra-low loss quantum photonic circuits integrated with single quantum emitters"

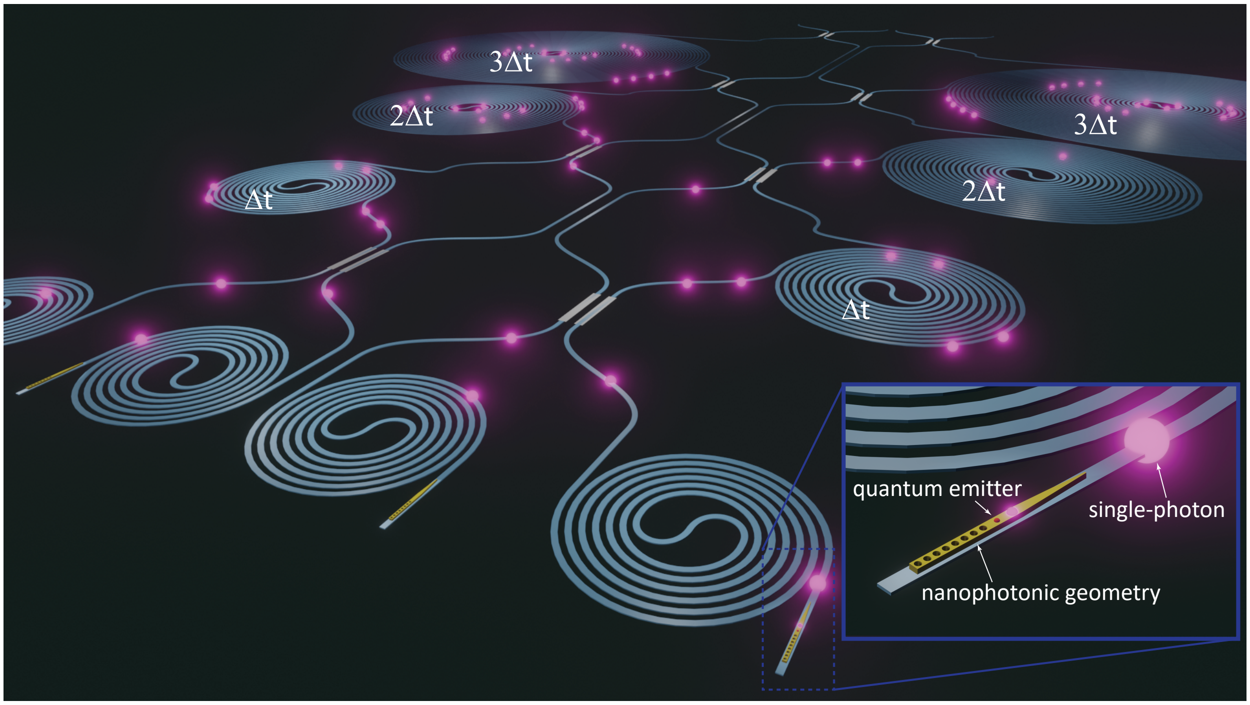

Supplementary Figure 1. Representation of a class of quantum photonic circuits enabled by the demonstrated ultra-low loss photonic integrated circuit platform. Here single-photons generated on-demand by single quantum emitters embedded in an optimized nanophotonic geometry (bottom right) are coupled into a photonic circuit composed of ultra-low loss waveguides. The ultra-low loss allows buffering and temporal multiplexing of emitted single-photons in on-chip spirals, a central capability for various fault-tolerant photonic quantum computing and simulation schemes [1–3] Δt here refers to spiral time-delays.

SUPPLEMENTARY NOTE 1 - LOW-LOSS INTEGRATED QUANTUM PHOTONIC PLATFORMS

Supplementary Table **1** shows the lowest reported propagation losses, to the best our knowledge, for various current integrated quantum photonic platforms. Also indicated are whether the demonstrated devices included on-chip sources, and of what type.

Supplementary Table 1. Reported propagation losses for various photonic integration platforms used in quantum photonics demonstrations to date. SFWM: spontaneous four-wave mixing; SPDC: spontaneous parametric down-conversion. PPLN: periodically-poled lithium niobate

**Integration platform**	**Losses (dB/m)**	**Wavelength (nm)**	**Source type**	**On-/off-chip source**	**Foundry-compatible***	**Reference**
Laser-written SiO_2	< 30	1550	no on-chip source	Off-chip	No	[4]
Laser-written SiO_2	≈ 50	800	no on-chip source	Off-chip	No	[5]
UV-written silica-on-Si	≈ 15	1550	SFWM	On-chip	No	[6]
Double-stripe Si_3N_4	≈ 10	1550	SPDC	Off-chip	Yes	[7]
Double-stripe Si_3N_4	≈ 20	1550	SFWM	On-chip	Yes	[8]
Hydex	≈ 5	1550	SFWM	On-chip	Yes	[9]
Si_3N_4 ridge	≈ 5 [a]	1550	SFWM	On-chip	Yes	[10]
Si_3N_4 ridge	≈ 20 [a]	1550	Squeezed light	On-chip	Yes	[11]
AlN ridge	≈ 100 [b]	1550	SPDC	On-chip	Yes	[12]
Silicon-on-insulator	≈ 100	1550	SFWM	On-chip	Yes	[13]
AlGaAs-on-Insulator	≈ 20	1550	SFWM	On-chip	Yes	[14]
Ti:PPLN	$\approx 1.6/\approx 2.2$	890 / 1320	SPDC	On-chip	No	[15]
Ti:$LiNbO_3$	≈ 10	1550	SPDC	Off-chip	No	[16]
Thin-film $LiNbO_3$	≈ 30	1550	SPDC	Off-chip	No	[17, 18]
III-V/Si_3N_4	$\approx$100	900	InAs quantum dot	On-chip	Yes	[19]
III-V/Si_3N_4	$\approx$250	900	InAsP quantum dot	On-chip	Yes	[20]
III-V/Si_3N_4	$\approx$ 1	900	InAs quantum dot	On-chip	Yes	**This work**

[a] Estimated from measured microring resonator intrisic quality factors, $Q_i \approx 7 \times 10^7$.

[b] Estimated from measured microring resonator intrinsic quality factors $Q_i \approx 4 \times 10^5$.

* Having at least passive photonic circuits that can be produced in a semiconductor foundry.

Our current work presents a hybrid integrated single quantum emitter to foundry-compatible Si_3N_4 integrated quantum photonic platform with propagation losses of $\approx$ 1 dB/m.

SUPPLEMENTARY NOTE 2 - SINGLE-PHOTON OPTICAL TIME-DOMAIN REFLECTOMETRY (SP-OTDR) SETUP

Supplementary Figure **2**(a) shows the schematic of the experimental setup used for our SP-OTDR measurements. An 80 MHz mode-locked fiber laser was used to produce < 100 fs pulses centered at a wavelength of $\approx$930 nm. An electro-optic modulator (EOM) synchronized to the laser was used to attenuate its output by > 20 dB, allowing a single pulse to pass at a period of more than 100 ns. This was done in an attempt to limit the observation of stray reflected pulses within the time-window corresponding to the spiral lengths. The modulated signal was passed through a 90:10 fiber splitter, then coupled to a lensed optical fiber connected to the 10 % port of the latter. The lensed fiber both launched the pulses into the on-chip ULLWs and collected the reflected light. The latter was then routed to superconducting nanowire single-photon detectors (SNSPDs) via the 90:10 splitter. The time correlator had a specified temporal resolution of 4 ps. Coupling into the TE Si_3N_4 waveguide mode was achieved by maximizing the reflected signal by varying the polarization of the injected light with a fiber polarization controller (FPC) placed before the 90:10 splitter. Minimizing the reflected signal (consistent with TM-polarization coupling) allowed us to obtain a mostly flat noise background, with spurious peaks which were also visible on the TE polarization curve. Such peaks originate from polarization-independent interfaces (e.g., fiber connectors and the chip facet) or scatterers along the probing light path, or are pulses that were insufficiently attenuated by the EOM. The TE mode and TM-polarization (background) reflectivity curves for the three measured spirals are shown in Fig. 3(b) in the main text. Subtracting the background signal from the TE reflectivity curves allowed us to reduce some of these features. The fits reported in Fig. 3(d) were done on such background-subtracted data, also displayed in the figure. To find the start of each spiral in time, we note that each spiral trace starts with a tall peak due to the chip facet. We select as the spiral start time the point at the onset of the facet peak, where the intensity is 10 dB higher than the preceding (background) intensity level.

The optical path traversed by the photons back-scattered at any point is twice the length of the spiral at the point. Thus the total length traversed by the photons, for calculation of loss, is twice the length of the spiral or the length shown as horizontal axis in Supplementary Figure **2**(b). Since the high-aspect ratio ULLW only supports the TE mode, the TM mode doesn't propagate in the waveguide and is reflected from the chip facet. Thus the OTDR for TM polarization also serves as the reference signal. The common peaks among the signal for TE and TM polarization, as well as

after other trains of pulses, indicates that these originate from elsewhere. These were identified as reflection from other optical components used in the setup.

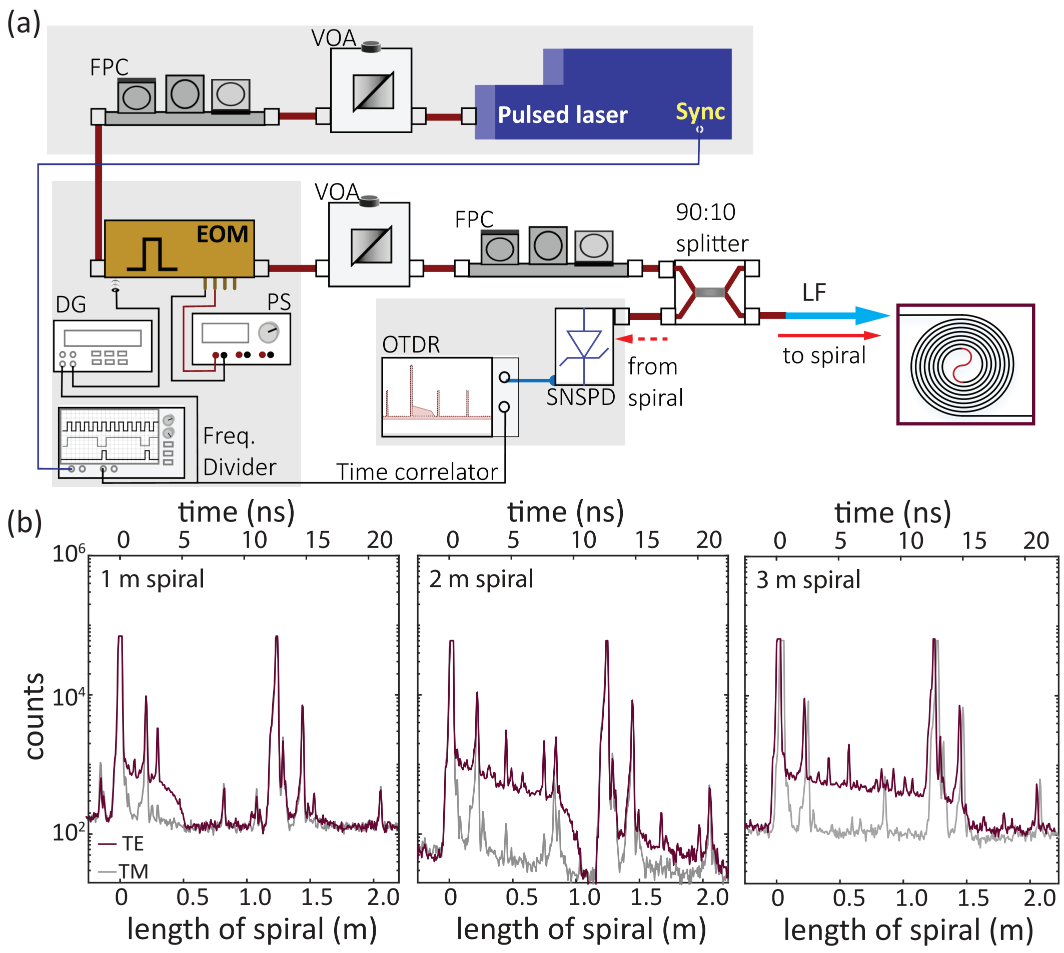

Supplementary Figure 2. Single-photon time-domain reflectometry setup. (a) Schematic of experimental setup for single-photon optical time-domain reflectometry (SP-OTDR) measurements. FPC: fiber polarization controller; VOA: variable optical attenuator; EOM: electro-optic modulator; PS: power supply; DG: delay generator. LF: Lensed Fiber. (b) SP-OTDR signal for back-scattered light from 3 m Archimedes spiral, upon injection of < 100 fs laser pulses. Exponentially decaying counts are observed as a function of time after pulse at zero delay for TE polarized excitation (red), indicating propagation in the spiral. Data for TM polarization is shown in gray, indicating much larger propagation losses, as to be expected given that the waveguide does not support a TM mode.

SUPPLEMENTARY NOTE 3 - DETERMINATION OF THE ULLW GROUP INDEX

The time-axis in our SP-OTDR measurements can be converted into a propagation length along the ULLWs though the relation

$$\Delta L = \frac{c_0 \Delta t}{2 n_g}, \tag{1}$$

where c_0 is the velocity of light in vacuum, ΔL is the length propagated in a time interval Δt, and n_g is the group index of the waveguide. To estimate n_g in the ULLWs, we performed SP-OTDR measurements on a straight waveguide fabricated on the same chip as the spirals studied in the main text. Supplementary Figure **3** shows time-dependent reflectivity traces obtained my maximizing and minimizing the insertion loss (IL) via the input polarization. Both traces were obtained with the same integration time of 60 s. Two peaks separated by ≈ 200 ps are apparent in the figure. Because the ULLW was designed to be close to single-mode, we assign the low IL (blue) curve to the fundamental transverse-electric (TE) mode, whereas the gray curve is assigned to the first transverse-electric (TM) mode. Because the first peaks for both the TE and TM polarizations have approximately the same amplitude, we assign such peaks to reflection at the waveguide facet, at the edge of the chip just after the lensed fiber. The next peaks, spaced by ≈ 200 ps, are assigned to the first reflection at output end of the ULLW. This assignment is based on the following reasoning.

A two-Gaussian fit to the TE polarization trace (continuous curve in Supplementary Figure **3**) gives a delay $\Delta t = (-206.0 \pm 0.9)$ ps, amplitude ratio $R_2/R_1 = 0.260 \pm 0.004$, and a width ratio $\sigma_2/\sigma_1 = 1.040 \pm 0.005$ between the first and second peaks. The uncertainties here are 95 % confidence intervals from the nonlinear fit, corresponding to two standard deviations. We note that the pulse widths are limited by the temporal resolution of our TCSPC system.

Assuming that the Gaussian beam produced by the lensed fiber refracts at the interface following simple Fresnel relations for normal incidence, the reflection coefficients at the first and second facets, r_1 and r_2, are

$$r_{1,2} = \pm \frac{n_o - n_i}{n_o + n_i}, \tag{2}$$

where n_i and n_o the refractive index outside and inside the device dielectric. We take $n_o = 1.0$ for air, and $n_i \approx n_{\text{eff}} = 1.466$, the TE mode effective mode index, computed with the Finite Element Method. The Fresnel transmission coefficients at the two facets are

$$t_{1,2} = 2 \frac{n_{o,i}}{n_o + n_i}. \tag{3}$$

The reflectance from the first facet back into the fiber is $R_1 = \eta_1 |r_1|^2$. We have introduced η_1 to

represent coupling losses between the free-space beam reflected at the facet and the fiber mode. The reflectance from the second facet back into the fiber is

$$R_2 = \eta_2^2 \left| r_2 t_1 t_2 \right|^2, \tag{4}$$

accounting for Fresnel transmission at the first facet, reflection at the second facet and transmission back through the first facet. The factor η_2 is introduced to represent modal coupling losses between the fiber and the TE waveguide mode. With this,

$$\frac{R_2}{R_1} = \frac{\eta_2^2}{\eta_1} \left| t_1 t_2 \right|^2 \approx 0.9 \frac{\eta_2^2}{\eta_1} \tag{5}$$

The fiber-to-ULLW coupling efficiency is estimated as $\eta_2 \approx 0.55$, as described in Section , from insertion loss measurements on straight waveguides fabricated on the sample chip as the spirals. Replacing in eq. (5), we obtain $R_2/R_1 \approx 0.27/\eta_1$. Comparing with $R_2/R_1 \approx 0.3$ obtained from the experimental fits above, we estimate $\eta_1 \approx 0.9$, which is a reasonable value.

Next, we used an optical microscope with an encoded sample stage to determine the length L of the measured waveguide. The stage encoder had a specified resolution of better than 100 nm. Using the coordinates of the four corners of the rectangular waveguide geometry at 100× magnification, we obtain $L \approx 20.9$ mm over four measurements. The Student t-distribution 95 % confidence interval for the four measurements is ≈ 0.6 μm. Using the $n_g = 1.51$ value computed with the finite element method, we calculate an expected delay $\Delta_t \approx 210$ ps between the first two reflected pulses, which is reasonably close to the ≈ 206 ps value obtained from the time-trace fit (i.e., it corresponds to a length difference of ≈ 1 mm, which we take to be our length uncertainty in Fig 3d of the main text). Taking the measured waveguide length and fitted delay between the pulses into eq. (1), we obtain $n_g = 1.482 \pm 0.006$. The uncertainty reported here is obtained by propagating uncertainties for Δt and L in eq. (1). For Δt, we use the 95 % fit confidence interval reported above as the uncertainty, whereas for L we use the Student t-distribution 95 % confidence interval.

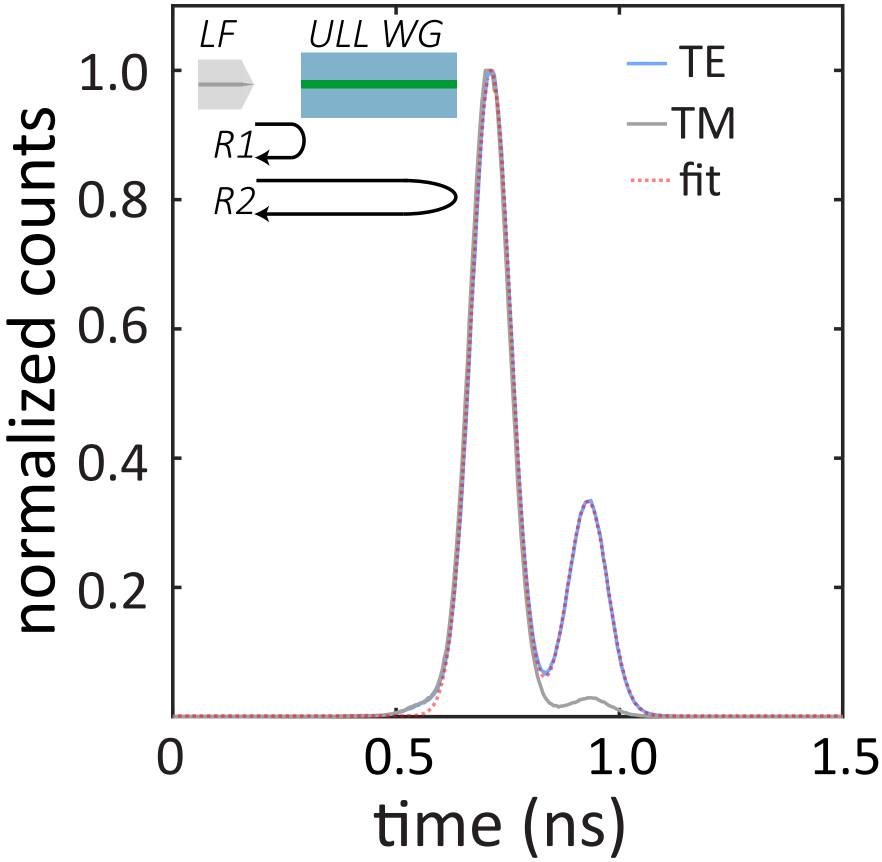

Supplementary Figure 3. Straight waveguide time-domain reflectometry. Experimental time-of-flight traces are shown for short pulses reflected from an ≈21 mm long straight ultra-low loss waveguide (ULLW), launched from a lensed optical fiber (LF) in endfire probing configuration. The two peaks correspond to first (R_1) and second (R_2) facet reflections, as indicated in the inset. Curves for TE and TM polarization are shown, evidencing considerably larger propagation losses for the latter, as expected. A fit to the TE curve is shown as a dotted line.

SUPPLEMENTARY NOTE 4 - IDENTIFICATION OF QUANTUM DOT EXCITONIC TRANSITIONS

As shown in Supplementary Figure **4**(a), we observed multiple QD emission lines upon non-resonant continuous-wave excitation at 841.4 nm, within $\approx$ 1.5 nm range, namely 926.02 nm, 926.57 nm and 927.21 nm, which could originate from multiple transitions of the same QD. To identify the excitonic species corresponding to each peak, we collected photoluminescence spectra for increasing excitation powers, as shown in Supplementary Figure **4**(b), and fitted the log-log curves to obtain their respective power-law coefficients. As indicated by the log-log slopes displayed on the power series plot, we observed almost linear (with a 1.05 slope) increase with excitation power for the 927.21 nm, which suggested a neutron exciton transition. The two higher energy peaks at 926.02 nm, 926.57 nm displayed superlinear increase with slopes $\approx$ 1.4 and $\approx$ 1.56, respectively. To probe further, we performed lifetime measurements on the three peaks, after filtering with two grating filters of $\approx$ 500 pm bandpass. Supplementary Figure **4** (c) shows the normalized radiative decay traces measured with pulsed 841.4 nm excitation at a saturation power of 55 μW. The lifetimes for the 926.02 nm, 926.57 nm and 927.21 nm lines, obtained from fits, are (0.754 ± 0.015) ns, (0.454 ± 0.013) ns and (1.06 ± 0.043) ns, respectively. The ratio of roughly two between the lifetimes of the 927.21 nm and 926.57 nm lines, together with the power-laws above, suggest that the two peaks correspond to the neutral exciton and biexciton states, respectively [21]. To validate, we measured the second-order cross-correlation, $g^{(2)}(\tau)$ between pairs of the three emission lines. As shown in Supplementary Figure **4**(d), asymmetric anti-bunching dips with $g^{(2)}(0) < 0.5$ are observed in all cases, confirming that all three transitions are from the same QD. However, none of the $g^{(2)}(\tau)$ curves exhibits the characteristic bunching expected from cascaded biexciton-exciton cascade [22, 23]. Basd on this, we assign the 927.21 nm peak to a neutral exciton (X_0), and the remaining two peaks to charged excitonic states (C_1 and C_2 for the 926.57 nm and 926.02 nm, respectively).

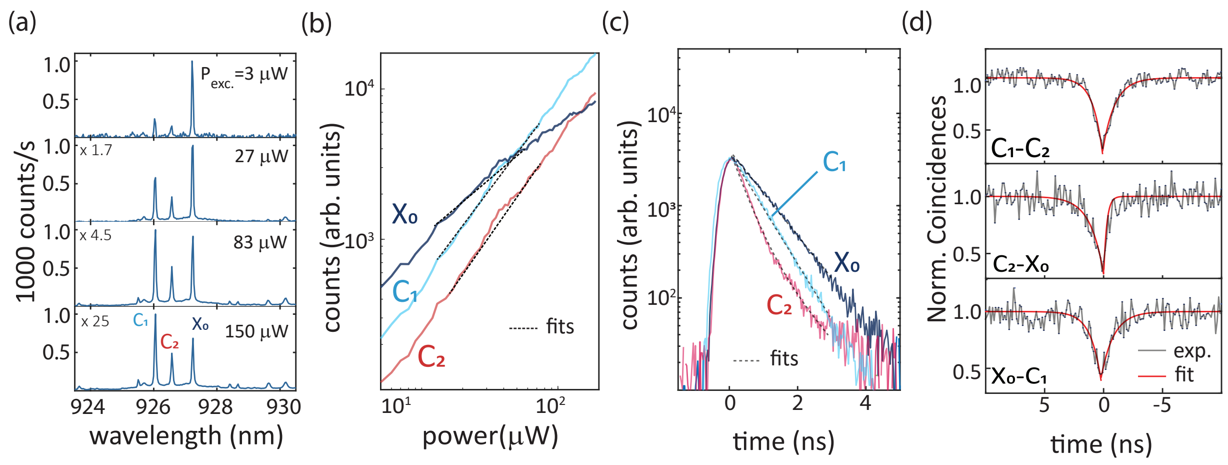

Supplementary Figure 4. Identification of single quantum dot transitions. (a) Photoluminescence spectrum, on non-resonant excitation at 841.1 nm, at different excitation powers $P_{exc.}$ showing relative change in intensity of three QD lines. (b) Peak intensity for the C_1 (light blue), C_2 (red) and X_0 (blue) lines plotted in log-log scale. Black Dashed lines are fits. (c) Radiative lifetime traces for the three lines. Black dashed lines are fits. (d) Second-order cross-correlation intensity traces between pairs of emission lines, C_1-C_2, C_2-X_0 and X_0-C_1. Experimental data is in black, red lines are fits. An antibunching dip at zero delay is seen in all cases, indicating that the three lines belong to the same emitter.

SUPPLEMENTARY NOTE 5 - SINGLE-PHOTON COUPLING EFFICIENCY

We estimate the single-photon coupling efficiency $\eta_{\text{QD-ULLW}}$ from the QD into the ULLW in our device by pumping the former into saturation with an 80 MHz pulsed laser at 880 nm, assuming 100 % quantum efficiency for the emission, and comparing with the single-photon detection rate, after taking into account all the losses along the path to the detectors. Specifically the ULLW-coupled QD light goes through the MMI with efficiency η_{MMI}, and is collected by the lensed fiber with efficiency $\eta_{\text{LF}} \cdot \eta_{\text{facet}}$. In the latter expression, the first factor is just due to the fiber itself, including the FC/APC connector it featured on one end, and the second the fiber-to-waveguide coupling efficiency at the facet. Fiber-coupled photons are subsequently passed though a ≈ 0.5 nm bandpass grating filter, with efficiency η_{filter}, then routed to an SNSPD with detection efficiency η_{SNSPD} via an optical fiber path with efficiency η_{fiber}.The detection rate at the SNSPDs is given by $R_{\text{det.}} = \eta_{\text{QD-ULLW}} \cdot \eta_{\text{MMI}} \cdot \eta_{\text{facet}} \cdot \eta_{\text{LF}} \cdot \eta_{\text{filter}} \cdot \eta_{\text{fiber}} \cdot \eta_{\text{SNSPD}}$.

We take the multimode interference (MMI) coupler efficiency to be $\eta_{\text{MMI}} < 0.445$, where the equality holds for the value determined from simulations. Figure **5** shows μPL spectra produced by one of the fabricated devices under 845 nm continuous wave (CW) laser pumping, collected individually from each of the MMI output ports. Identical spectral features and comparable photon counts at the two ports suggest that the designed MMI 50 : 50 split ratio is within reach.

The grating filter efficiency η_{filter} was estimated to be ≈ 45 % based on QD spectra taken before and after the filtering. The lensed fiber insertion loss was estimated by injecting CW laser light into it by way of the 10 % port of a 90:10 fiber beam splitter and measuring the reflected power, after positioning the lensed fiber below the lower SiO_2 cladding on the chip. In this case, light launched into free-space by the lensed fiber was reflected at the polished Si substrate facet and coupled back into the former. Assuming only Fresnel reflection at the air-to-Si interface, and perfect coupling back into the lensed fiber, $\eta_{\text{LF}} \approx 0.5$. It is likely, however, that the fiber coupling efficiency for the reflected free-space light was not perfect, so this estimate is at best a lower bound. For an upper bound, we take simply the 0.25 dB insertion loss that is specified for the FC/APC mating sleeve.

Facet losses η_{facet} were estimated from insertion losses measured from three $\approx$ 20 mm long straight ULLWs fabricated on the same chip as the Archimedean spirals described in the main text. In such short straight guides, propagation losses amount to $<$ 3 dB/m$\times$0.02 m $<$ 0.1 dB, so we estimate η_{facet} as half the insertion loss. From all three waveguides, we obtain $\eta_{\text{facet}} = 0.55 \pm 0.02$, where the uncertainty corresponds to the standard deviation among all measured values. We note

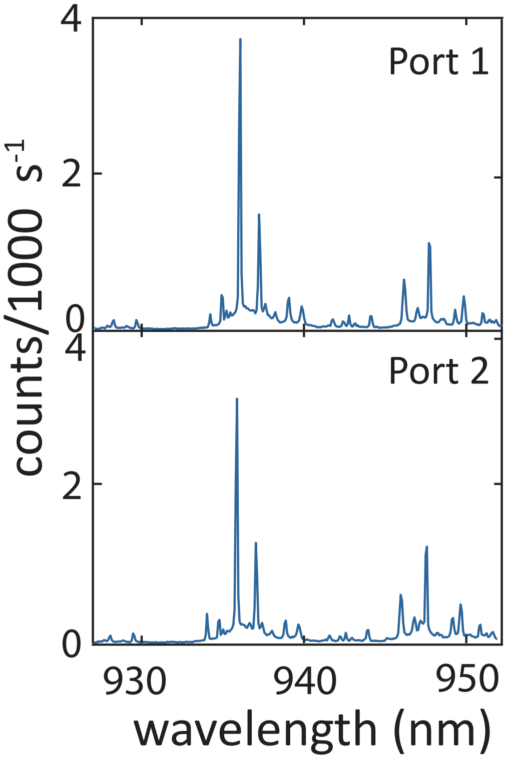

Supplementary Figure 5. On-chip directional splitting of quantum dot emission. Photoluminescence collected separately from the two output ports of a ULLW based 50:50 MMI splitter is shown for QD pumping at 845 nm.

that simulations had originally predicted a coupling efficiency of ≈ 0.63 dB between a ≈ 2.0 μm Gaussian spot and a ULL WG of width 2 μm. The discrepancy between measured and simulation values is likely due to modal mismatch with the actual tapered optical fiber that was utilized.

The fiber that linked the output of the grating filter to the input of the SNSPD was measured to have a transmission of ≈ 90 %. Finally, we estimated the SNSPD efficiency $\eta_{\text{SNSPD}} = 0.71 \pm 0.03$ by noting the count rates obtained from a calibrated laser signal at the QD wavelength. The uncertainty here was propagated from power and count rate measurement uncertainties due to experimental fluctuations.

For a detected SNSPD count rate of $\approx 2.1 \times 10^5 \text{s}^{-1}$, we find, using the MMI efficiency upper bound $\eta_{\text{MMI}} = 0.445$, the QD-ULLW coupling $\eta_{\text{QD-ULLW}} = R_{\text{det.}}/80$ MHz to be within a 4 % to 7 % interval. The uncertainty here is largely due to the lensed fiber insertion loss η_{LF}. Since however we expect $\eta_{\text{MMI}} \leq 0.445$, then $\eta_{\text{QD-ULLW}} \geq 4$ %, conservatively.

As shown in Section of the SI, the QD dipole moment orientation and position have the largest impact on the overall coupling efficiency $\eta_{\text{QD-ULLW}}$. Since neither position nor orientation of the accessed QD were known or controlled, it is likely that these two factors account for the relatively low observed $\eta_{\text{QD-ULLW}}$.

SUPPLEMENTARY NOTE 6 - COUPLING EFFICIENCY SIMULATIONS

To assess the expected QD-to-ULLW coupling efficiency $\eta_{\text{QD-ULLW}}$, we performed FDTD simulations of an electric point dipole source radiating inside of a hybrid waveguide geometry that approximates that of the tested device. In particular, we attempt to include geometrical imperfections that were apparent from the scanning electron micrograph in Fig. 2(b) of the main text, as well as the QD location and dipole moment orientation with respect to the GaAs host nanowaveguide. Supplementary Table **2** shows the coupling efficiency $\eta_{\text{QD-ULLW}}$ and Purcell factors F_p calculated considering combined variations of $\theta_{\text{dip.}}$, the dipole orientation with respect to the GaAs waveguide axis; $\Delta x_{\text{dip.}}$, the dipole displacement from the GaAs WG center; Δx_{WG}, the lateral displacement between the GaAs and Si_3N_4 WGs; and $\Delta\theta$, the angular misalignment between the Si_3N_4 and GaAs waveguides. The first row of the Supplementary Table **2** corresponds to the ideal case.

Supplementary Table 2. Simulated dipole quantum efficiency $\eta_{\text{QD-ULLW}}$ and Purcell factor F_p into the ultra-low loss waveguide for various geometrical parameters defined in the text.

$\theta_{\text{dip.}}(^\circ)$	$\Delta x_{\text{dip.}}$ (nm)	Δx_{WG} (nm)	$\Delta\theta(^\circ)$	F_p	$\eta_{\text{QD-ULLW}}$
90	0	0	0	2.01	0.31
90	0	500	0	1.96	0.22
90	67.5	0	0	1.27	0.28
90	0	340	0.9	2.06	0.30
90	67.5	340	0.9	1.26	0.27
0	0	0	0	0.87	0
0	67.5	0	0	0.52	0.001
70	0	0	0	1.02	0.073

It is apparent in these results that dipole orientation has the strongest influence in the coupling efficiency, primarily because longitudinally oriented dipoles ($\theta_{\text{dip.}} = 0$ with respect to the GaAs waveguide) does not couple to the (single) TE mode of the GaAs guide. Other geometrical imperfections contribute considerably less, within ≈ 30 %, to a decreased overall coupling efficiency. The Purcell factor F_p is affected almost exclusively by the position and orientation of the quan-

tum dot within the GaAs nanowaveguide. Taken altogether, these results indicate that sub-optimal position and orientation of the (non-deterministically positioned) quantum dot are likely the main contributors to the low observed coupling efficiency.

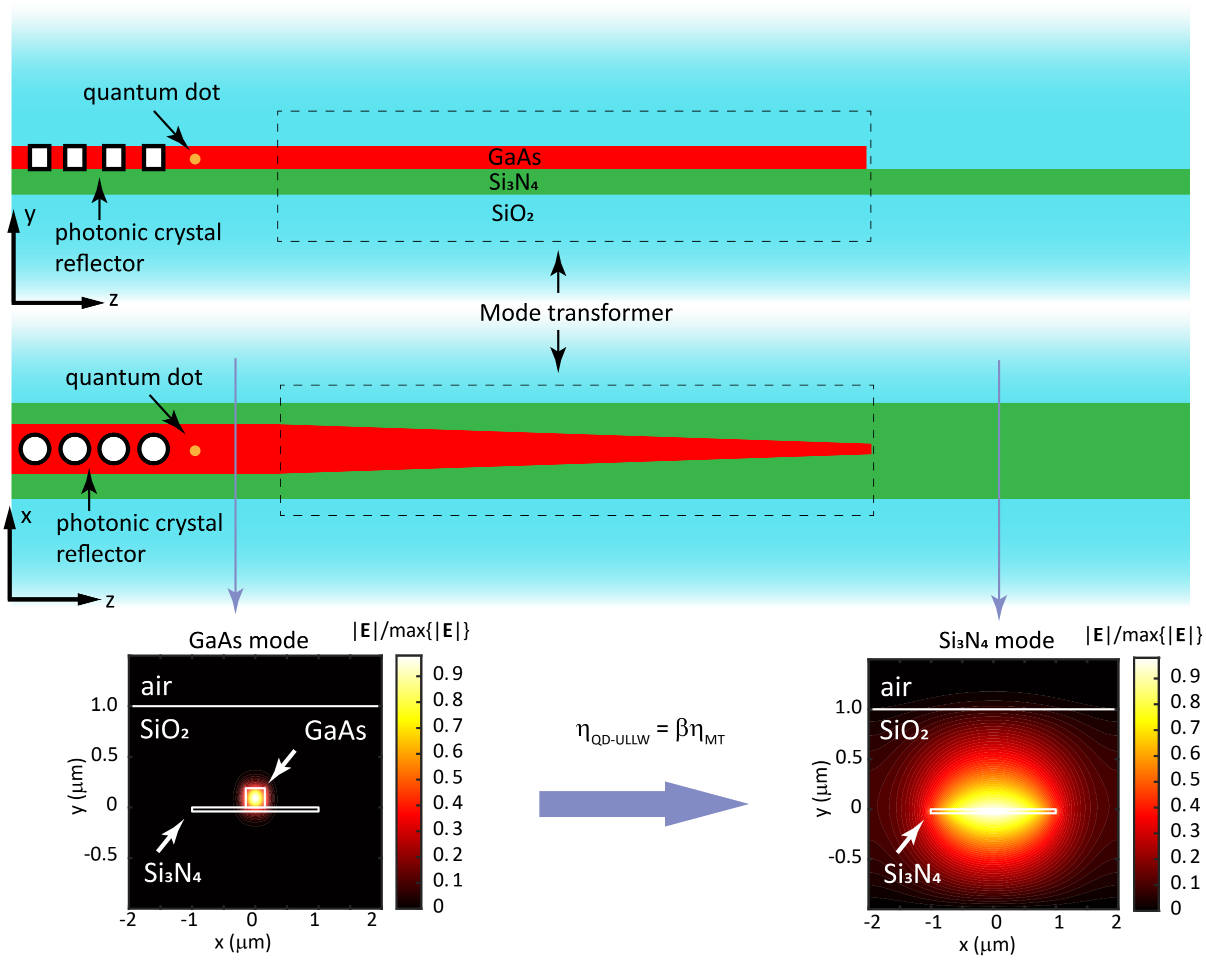

Supplementary Figure 6. Schematic of the implemented nanophotonic single-photon source. The quantum dot's emission is coupled with efficiency β into the GaAs mode (bottom left), whereas the mode transformer converts the GaAs mode into the Si_3N_4 mode (bottom right) with efficiency η_{MT}. Overall, the quantum dot coupling efficiency to the Si_3N_4 waveguide is $\beta \cdot \eta_{MT} = \eta_{QD\text{-}ULLW}$. The modal plots show the normalized mode electric field amplitude, **E**, normalized by its maximum value.

The maximum 31 % coupling efficiency can be broken down into two components, as indicated in Supplementary Figure **6**. A maximum $\beta \approx 88$ % QD coupling efficiency was predicted into the straight GaAs waveguide section of the light source. The GaAs mode transformer leading to the Si_3N_4 waveguide, featuring an unoptimized linear width taper, was predicted to have only

$\eta_{MT} \approx 35$ % efficiency. Overall, a maximum source efficiency of $\beta \cdot \eta_{\mathrm{MT}} \approx 31$ % could be expected from the implemented geometries.

SUPPLEMENTARY NOTE 7 - OPTIMIZED ADIABATIC TAPER DESIGN

Although our fabricated devices featured adiabatic couplers with relatively low (≈ 30 %) efficiency, below we assert the potential of our device platform regarding efficient single-photon emission into the ULLWs. We do so by designing a mode converter with > 93 % efficiency, following the procedure outlined in ref. [24], which defines an adiabaticity criterion based on a desired level of coupling loss for a minimized length. The latter characteristic is highly important to ensure stability of free-standing GaAs devices after removal of the AlGaAs sacrificial layer. We start by calculating the effective coupling length $L_{\mathrm{eff}} = \lambda/(n_1 - n_2)$ between the first two supermodes of the hybrid waveguide shown in the inset of Supplementary Figure **7**, as a function of the GaAs ridge width, w_{GaAs}. Here, $\lambda = 920$ nm is the design wavelength, and n_1 and n_2 are the supermode effective indices of the $\mathrm{TE}_{00}^{\mathrm{GaAs}}$ and $\mathrm{TE}_{00}^{\mathrm{Si_3N_4}}$ modes indicated in the figure. The effective length plotted in Supplementary Figure **7** varies considerably with w_{GaAs}, and indicates the necessary length scale for achieving adiabaticity in transitioning from the hybrid to the ULLW.

To determine the GaAs taper width profile $w_{\mathrm{GaAs}}(z)$ that minimizes coupling to the second-order supermode in the shortest length, we start from the equation [24]

$$\frac{1}{2\kappa(1+\gamma^2)^{3/2}} \frac{d\gamma}{dz} \leq \sqrt{\varepsilon}, \tag{6}$$

where ε is the (desired) power fraction that is coupled into the (unwanted) second supermode. In this expression, $\gamma = \delta/\kappa$, with $\delta = 2\pi(n_{\mathrm{eff}}^{\mathrm{GaAs}} - n_{\mathrm{eff}}^{\mathrm{Si_3N_4}})/\lambda$, is the propagation constant mismatch for the two individual, uncoupled waveguides, and $\kappa^2 = (S^2 - \delta^2)$, with $S = 2\pi(n_1 - n_2)/\lambda$, is the coupling strength between the two. Both parameters γ and κ are calculated as a function of w_{GaAs}, so that we can write

$$\frac{1}{2\kappa(1+\gamma^2)^{3/2}} \frac{d\gamma}{dw_{\mathrm{GaAs}}} \leq \sqrt{\varepsilon} \frac{dz}{dw_{\mathrm{GaAs}}}. \tag{7}$$

Integrating eq. (7) over a sufficiently small width variation δw_{GaAs} that the first factor on the left hand-side can be assumed to be constant, we obtain the local adiabatic coupling length $\delta z(w_{\mathrm{GaAs}})$

$$\delta z(w_{\mathrm{GaAs}}) = \frac{1}{2\sqrt{\varepsilon}\kappa(1+\gamma^2)^{3/2}} \delta\gamma(w_{\mathrm{GaAs}}), \tag{8}$$

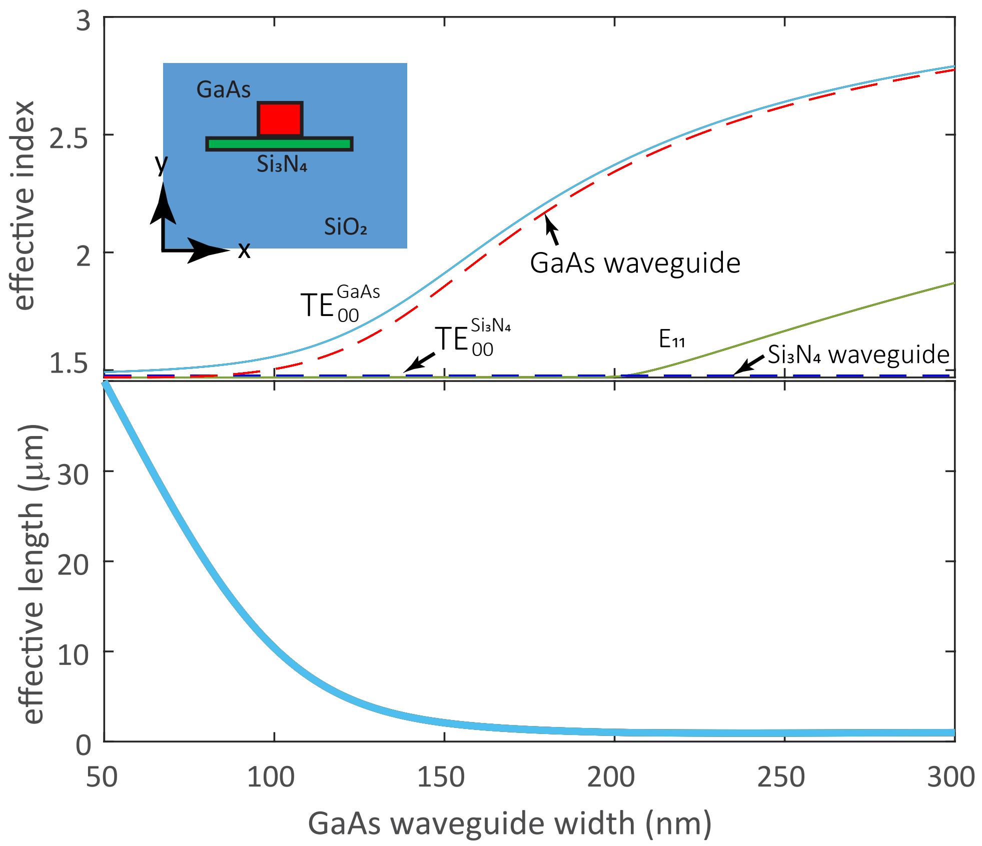

Supplementary Figure 7. Hybrid waveguide supermode calculations. Top: Continuous lines correspond to the effective indices of the supermodes of the hybrid waveguide geometry shown in the inset, as a function of GaAs waveguide width. Dashed lines are effective indices for the GaAs and Si_3N_4 waveguides taken alone. The GaAs and Si_3N_4 thicknesses were 190 nm and 40 nm respectively, and the Si_3N_4 width was 2 μm. The first two supermodes, labeled TE_{00}^{GaAs} and $TE_{00}^{Si_3N_4}$, are considered for the adiabatic taper calculation. A second-order hybrid mode, labeled E_{11}, appears at a GaAs width of $\approx$200 nm. Bottom: Effective adiabatic coupling length L_{eff} for the TE_{00}^{GaAs} and $TE_{00}^{Si_3N_4}$ modes, as a function of GaAs waveguide width.

which we then numerically integrate to obtain an optimized GaAs width profile. Optimized width profiles for our geometry starting from $w_{GaAs} = 200$ nm are shown in Supplementary Figure **8**(a), for $\varepsilon = 0.1$ and $\varepsilon = 0.01$. The conversion efficiencies for the two profiles, as a function of wavelength, are shown in Supplementary Figure **8**, calculated in finite difference time domain simulations in which the GaAs waveguide is excited with its fundamental TE mode at 920 nm. Supplementary Figure **8**(c) shows the adiabatic taper profile used in the simulation of the $\varepsilon = 0.01$ case.

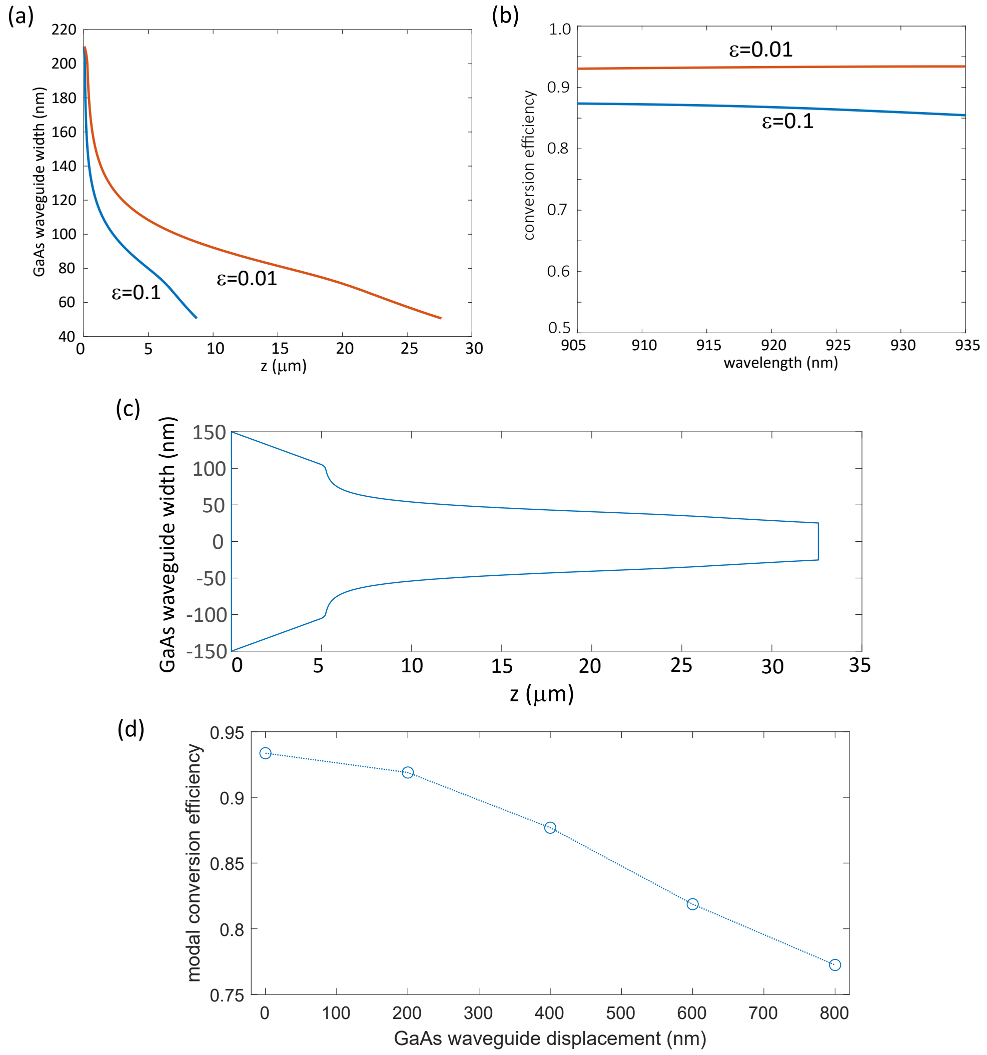

Supplementary Figure 8. Simulation of optimized mode transformers. (a) Optimized adiabatic GaAs width taper profiles for $\varepsilon = 0.1$ and $\varepsilon = 0.01$, whee ε is the power fraction that is coupled into the second supermode. (b) Simulated conversion efficiencies, from the TE_{00}^{GaAs} mode to the fundamental TE Si_3N_4 waveguide mode as a function of wavelength. (c) GaAs waveguide width profile obtained for $\varepsilon = 0.01$, used to obtain the simulated result in (b). (d) Mode transformer efficiency, calculated with FDTD, as a function of lateral GaAs waveguide displacement from the center of the Si_3N_4 waveguide. Circles are calculated values, lines a guide to the eye.

The relative placement tolerance of the GaAs taper over the Si_3N_4 can be appreciated in the FDTD simulation of modal transformation efficiency as shown in Supplementary Figure8(d). It is apparent that, even for a ≈ 400 nm displacement, a drop in efficiency of < 10 % can be expected.

SUPPLEMENTARY NOTE 8 - OPTIMIZED GAAS WAVEGUIDE-COUPLED SOURCE

Multi-objective inverse-design [25] was performed to produce an optimized GaAs source geometry for improved coupling efficiency. The optimization procedure sought to maximize simultaneously the Purcell factor F_p experienced by the emitter dipole inside the geometry, and the dipole coupling efficiency β into a GaAs waveguide of thickness 190 nm and width 300 nm embedded in SiO_2, same as for the fabricated devices,

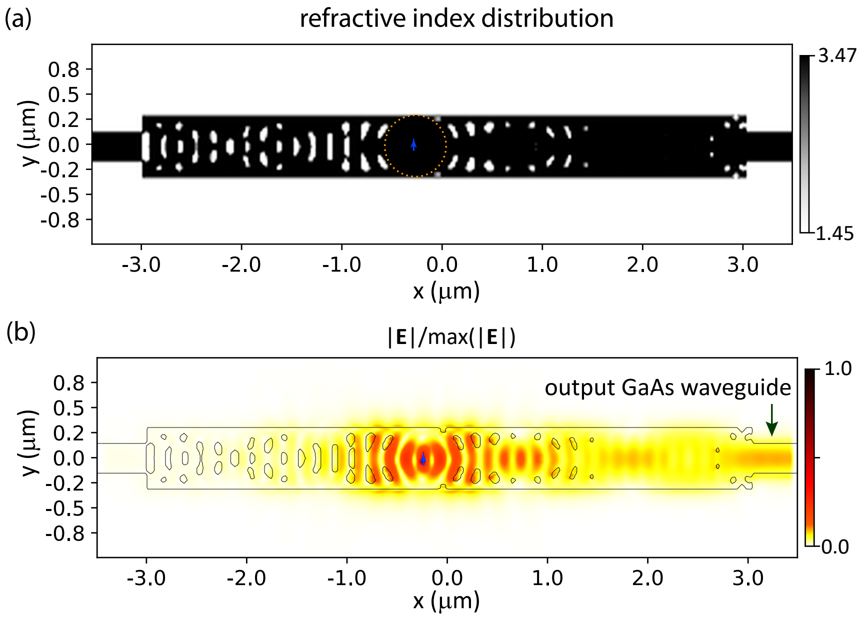

Supplementary Figure 9. Optimized GaAs cavity embedded in SiO_2, generated via multi-objective inverse design [25]. (a) Refractive index distribution at the z=0 plane. The dashed line indicates a circular region of radius 300 nm, centered at the quantum dot, represented by a blue arrow, where etched sidewalls are absent. The blue arrow indicates the optimal (y) quantum dot electric dipole moment orientation. (b) Normalized electric field (**E**) profile generated by a y-oriented dipole at the cavity center.

The optimized geometry, the cavity shown in Supplementary Figure **9**(a), supports the mode shown in Supplementary Figure **9**(b) that has $F_p \approx 10$ and $\beta \approx 0.91$. Importantly, the cavity features no etched sidewalls at distances less than 300 nm from the QD. Together with the relatively high F_p this characteristic has the potential to improve photon indistinguishability. In combination with the optimized mode transformer designed in Supplementary Note , Si_3N_4 waveguide coupling efficiencies of ≈ 85 % could be achieved. Further improvements may be possible.

SUPPLEMENTARY NOTE 9 - FITS FOR RESONANCE FLUORESCENCE SPECTRUM

As described in the main text, the measured Mollow triplet spectra, shown in Fig. 4c in the main text, presented slight asymmetries that suggested laser detuning [26] and spectral diffusion [27]. To obtain a better understanding of such features, we fit the data with the physical model derived in Konthasinghe *et al.* [27], which extends Mollow's original results for the case of nonzero laser detuning from the transition. The model in addition assumes that the transition spectrally wanders at time-scales longer than the radiative decay time T_1, corresponding to a time-varying detuning which is imprinted in the spectrum via the convolution

$$I(\nu,\Delta\omega) \propto \int \tilde{g}(\nu,\Delta\omega) \exp\left(-\frac{\Delta\omega^2}{2\sigma_{\mathrm{SD}}^2}\right) d\Delta\omega. \tag{9}$$

Here, $I(\nu,\Delta\omega)$ is the final spectrum, $\tilde{g}(\nu,\Delta\omega)$ is the detuning-dependent Mollow triplet spectrum [26, 27], $\Delta\omega$ is the laser-transition detuning, and σ_{SD} is a measure of the extent of the spectral diffusion, which is postulated to follow a Gaussian distribution. The coherently scattered laser and residual pump have been included in the fit model as a single resolution-limited Lorentzian peak. Supplementary Figure **10**(a) shows fits to the spectra obtained with the parameters in Supplementary Figure **10**(b), for the corresponding excitation powers P. As expected the Rabi frequencies Ω_{R} increase linearly with $\sqrt{P}$. The coherence time is seen to vary somewhat for varying powers, though remaining below 100 ps throughout. The spectral diffusion distribution full-width at half maximum, $\mathrm{FWHM}_{\mathrm{SD}}$, is seen to vary between 15 GHz and 20 GHz, except for the lower power, where the confidence interval is large. To understand whether such a large spectral diffusion is reasonable, we show, in Supplementary Figure **10**(c) a graph of the resonance fluorescence intensity as a function of laser detuning from the X_1 QD transition. In this measurement, low-resolution resonance fluorescence spectra were recorded with a grating spectrometer, and a wavelength meter with a 600 MHz nominal accuracy was used to record the laser frequency. As described in the Methods, an above-GaAs-band co-pump laser was used to optically gate the resonance fluorescence emission [28]. The plotted data correspond to the difference in integrated counts obtained with the co-pump on and off. The experimental data obtained in this fashion (red dots in Supplementary Figure **10**(c)) could be fit with a Gaussian with an FWHM $\approx$ 11 GHz, which is comparable to spectral diffusion FWHMs obtained from the Mollow triplet fits.

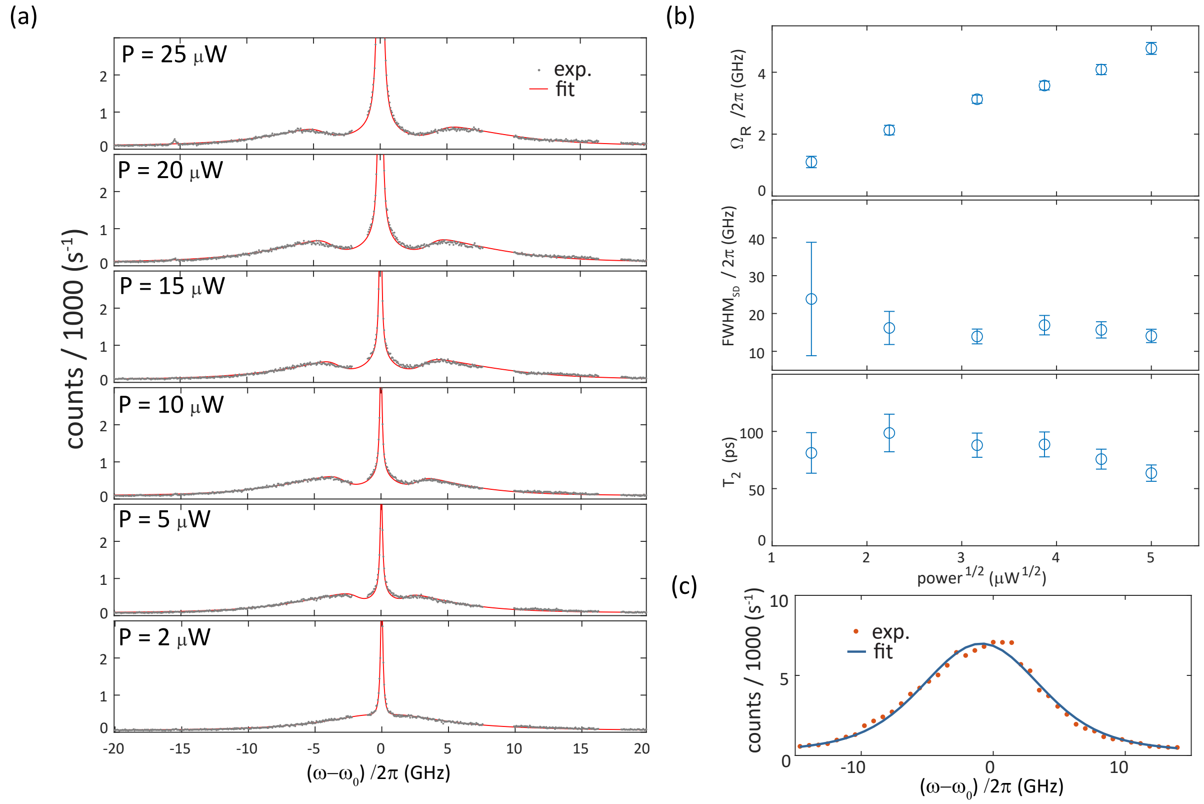

Supplementary Figure 10. Fits to resonance fluorescence data. (a) High-resolution resonance fluorescence spectra obtained with a scanning Fabry-Perot resonator (grey dots) and fit (red lines) using the Mollow triplet model of ref. 27, for varying nominal excitation power P. ω and ω_0 are the laser and quantum dot transition frequencies respectively. (b) Fit parameters as a function of the square-root of the excitation power. Ω_R: Rabi Frequency; FWHM$_{\mathrm{SD}}$: Gaussian spectral diffusion FWHM; T_2: coherence time. Uncertainties are 95 % fit confidence intervals, corresponding to two standard deviations. (c) Resonance fluorescence intensity as a function of laser detuning from the QD transition, corresponding to excitation laser power of 10 μW, extracted from low-resolution spectra from a grating spectrometer, as described in the text. Red dots: experimental data; continuous line:Gaussian fit.

SUPPLEMENTARY NOTE 10 - MODELLING OF INTERFEROMETRIC CORRELATION SPECTROSCOPY

We used a variable delay Mach-Zehnder interferometer to measure the correlation, $g^{(1)}$, of the filtered QD emission. To model the visibility of the interference fringes for QD emission under resonant excitation, we used the expression for the first-order correlation function [29]:

$$g^{(1)}(\tau) = e^{-\omega_L|\tau|}.\frac{1}{2}e^{-\frac{|\tau|}{T_L}}.\left[\frac{\frac{T_2}{2T_1}}{1+\Omega_{\mathrm{R}}^2 T_1 T_2} + \frac{E}{2}e^{-\frac{|\tau|}{T_2}} + Ge^{-\frac{1}{2}\left(\frac{\tau}{T_2}\right)^2} + \left(\frac{E}{2}e^{\eta|\tau|} + Ge^{-\frac{1}{2}\left(\eta\tau\right)^2}\right).\left[\alpha\cos(\nu\tau) + \beta\sin(\nu\tau)\right]\right] \quad (10)$$

Here,

$$\eta = \frac{1}{2}\left[\frac{1}{T_1} + \frac{1}{T_2}\right] \qquad \nu = \sqrt{\Omega_{\mathrm{R}}^2 + \frac{1}{4}\left[\frac{1}{T_1} - \frac{1}{T_2}\right]^2}$$

$$\alpha = 1 - \frac{T_2}{T_1(1+\Omega_{\mathrm{R}}^2 T_1 T_2)} \qquad \beta = \frac{\Omega_{\mathrm{R}}^2 T_1(3T_2 - T_1) - \frac{(T_1-T_2)^2}{T_1 T_2}}{2\nu T_1(1+\Omega_{\mathrm{R}}^2 T_1 T_2)}$$

The parameter T_L represents the coherence time of laser, which was estimated to be 20 μs based on the specified laser linewidth, and Ω_{R} is the Rabi frequency. The visibility traces were fit to estimate T_2 and Ω_R, while the measured value of $T_1 = (0.63 \pm 0.01)$ ns was used.

In eq. (10), the first term corresponds to a coherent laser background due to incomplete pump suppression which captures interference between laser fields and QD emission fields at resonant excitation. The term in parenthesis corresponds to exponential decay associated with coherence lifetime and oscillatory terms corresponding to the system undergoing Rabi cycles[30]. In addition, we consider coefficients of the two terms as a statistical sum of exponential and Gaussian monotonous decay, given by coefficients E and G, respectively, accounting for spectral diffusion of the QD.

Supplementary Figure **11** shows the visibility plot (black) for CW laser which was used to characterize the setup response accounting for imperfections in alignment and overlay of the two arms of the MZI. The curve was used as a reference to normalize the visibility traces for QD emission. Experimental data (red dots) and the corresponding fits to the model (blue) shows a representative trace for $g^{(1)}$ for resonance fluorescence in the strong drive regime, where Rabi oscillations are observed. The non-zero visibility obtained from fringe data at longer time-delay

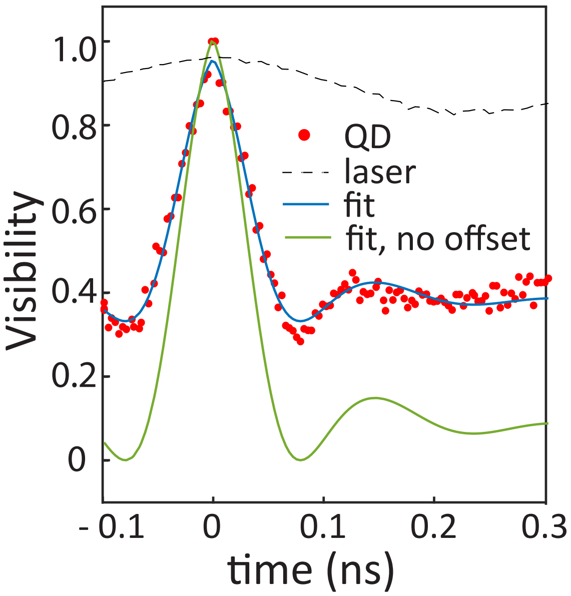

Supplementary Figure 11. Fitting interferometric correlation spectroscopy traces. Mach-Zehnder interferogram visibility (red points) of QD emission when excited resonantly and the corresponding fit (blue curve), normalized to the system response measured with laser as the MZI input (dashed black curve). The offset in the fit model accounts for the resonant laser that is not sufficiently suppressed. The green curve shows the normalized MZI visibility for the same parameter derived from the fit but without the offset.

($>$ 0.3 ns) originates from laser background. The trace in green shows the fitted function without the laser background.

Supplementary Table **3** shows the fit parameters for MZI visibility traces shown in Fig. 5c and 5d of the main text. To evaluate the relative importance of the Gaussian and Exponential components, their respective amplitudes, E and G, are displayed, normalized by E+G. At low Rabi frequencies, the visibility curve is well fitted by a two-sided exponential, while at higher powers the fit is predominantly Gaussian. In comparison, for p-shell excitation the fit has comparably relevant Gaussian and exponential components.

Supplementary Table 3. Fitted parameters for Gaussian and two-sided exponential for MZI visibility traces

**Excitation type**	**Exponential (E)**	**Gaussian (G)**
$\Omega_R/2\pi \approx 2$ GHz (Fig. 5d i)	≈ 0.91	≈ 0.09
$\Omega_R/2\pi \approx 7$ GHz (Fig. 5d ii)	≈ 0.12	≈ 0.88
$\Omega_R/2\pi \approx 11$ GHz (Fig. 5d iii)	≈ 0.05	≈ 0.98
p-shell (5e)	≈ 0.62	≈ 0.38

SUPPLEMENTARY NOTE 11 - TRIGGERED SINGLE PHOTON EMISSION UNDER RESONANT EXCITATION

We measured QD emission under pulsed resonant excitation, from which we extract the radiative lifetime and demonstrate triggered single photon emission. Pulsed measurements were done using an 80 MHz, < 100 fs pulse train at the resonant wavelength of the X_0 transition and an additional weak non-resonant laser at ≈ 841 nm that limits QD occupation with dark (non-radiative) states, thereby increasing emission counts (the intensity of the red laser on its own is not enough to produce appreciable QD emission). The resonant laser scattering was minimized by varying the polarization of the excitation laser. Supplementary Figure **12**(a) shows radiative decay trace fit to exponential decay. The radiative lifetime is $\tau_1 = (0.63 \pm 0.01)$ ns. To determine the purity of single photons under pulsed resonant excitation, the second-order (intensity) correlation, $g^{(2)}(\tau)$, was measured using a Hanbury-Brown and Twiss arrangement employing two SNSPDs.

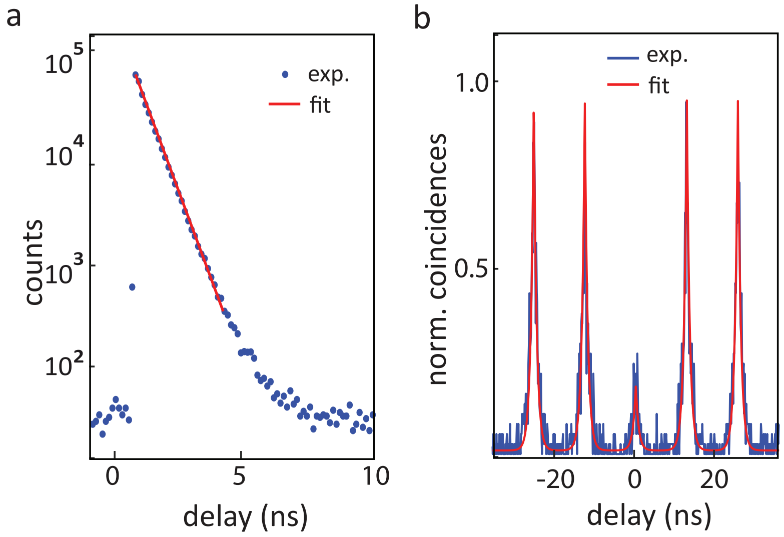

Supplementary Figure 12. Single-photon emission characteristics under resonant excitation. (a) Radiative decay trace and fitted exponential decay. The radiative lifetime under resonant excitation, τ_1, was measured to be(0.63 ± 0.01) ns. (b) Second-order intensity correlation for exciton emission showing triggered single photon emission under resonant excitation, with fitted $g^{(2)}(0) = 0.18 \pm 0.03$.

Supplementary Figure **12**(b) shows the normalized coincidence counts and fit to two-sided exponential decays. The fitted values were obtained as $g^{(2)}(0) = 0.18 \pm 0.03$ and decay parameter of (0.67 ± 0.03) ns (the latter is consistent with the directly measured excited state lifetime in Supplementary Figure **12**(a)). The value of $g^{(2)}(0)$ below 0.5 shows triggered single photon emission under resonant excitation.

SUPPLEMENTARY NOTE 12 - COMPARISON OF EXCITON LINEWIDTH UNDER NON-RESONANT AND RESONANT EXCITATION

To qualitatively compare the photon indistinguishability for the QD under non-resonant and resonant excitation, we measured QD emission linewidths with a scanning Fabry-Pérot interferometer. For non-resonant excitation, the QD was excited at wavelength of 848.89 nm, corresponding to the wetting layer, and at 887.90 nm, corresponding to the QD p-shell. The spectra are shown in Supplementary Figure. **13** (a) and (b), respectively. In the figure, the data was fit to a Voigt lineshape, with $\Delta\nu_G$ and $\Delta\nu_L$ the Gaussian and Lorentzian component linewidths, respectively. In comparison, Supplementary Figure **13** (c) shows the emission spectrum under resonant excitation. Here, the scattered resonant laser background, obtained with above-band gating laser off, has been subtracted. The resonance fluorescence data here was fit with a Lorentzian function for the central peak, and Gaussians for the sidebands.The extracted linewidth for the central Lorentzian peak is $\Delta\nu_{L-RF} = (0.67 \pm 0.04$ GHz), considerably narrower than the non-resonant illumination linewidths.

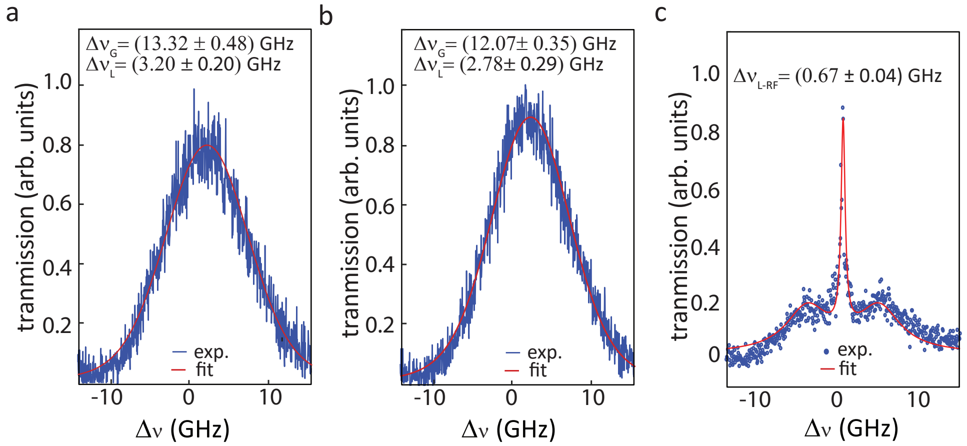

Supplementary Figure 13. Comparison of emission spectra for different excitation types. Scanning Fabry-Pérot interferometer traces across QD emission when excited non-resonantly (a) at wavelength of 848.89 nm and (b) at wavelength of 887.90 nm. Experimental data is plotted in blue, whereas red curves are fits using a Voigt lineshape with Gaussian and Lorentzian linewidths $\Delta\nu_G$ and $\Delta\nu_L$ respectively. (c) Corresponding trace at resonant excitation showing RF spectrum. $\Delta\nu_{L-RF}$ is the Loretzian linewidth used to fit the central peak.All uncertainties are 95 % confidence intervals from fits, corresponding to two standard deviations.

SUPPLEMENTARY REFERENCES

1. Gimeno-Segovia, M. *et al.* Relative multiplexing for minimising switching in linear-optical quantum computing. *New Journal of Physics* **19**, 063013 (2017). URL https://doi.org/10.1088/1367-2630/aa7095. Publisher: IOP Publishing.
2. Brod, D. J. *et al.* Photonic implementation of boson sampling: a review. *Advanced Photonics* **1**, 034001 (2019). URL https://www.spiedigitallibrary.org/journals/advanced-photonics/volume-1/issue-3/034001/Photonic-implementation-of-boson-sampling-a-review/10.1117/1.AP.1.3.034001.full. Publisher: SPIE.
3. Deshpande, A. *et al.* Quantum computational advantage via high-dimensional Gaussian boson sampling. *Science Advances* (2022). URL https://www.science.org/doi/abs/10.1126/sciadv.abi7894.
4. Ceccarelli, F. *et al.* Low Power Reconfigurability and Reduced Crosstalk in Integrated Photonic Circuits Fabricated by Femtosecond Laser Micromachining. *Laser & Photonics Reviews* **14**, 2000024 (2020). URL https://onlinelibrary.wiley.com/doi/abs/10.1002/lpor.202000024. _eprint: https://onlinelibrary.wiley.com/doi/pdf/10.1002/lpor.202000024.
5. Spagnolo, N. *et al.* Experimental validation of photonic boson sampling. *Nature Photonics* **8**, 615–620 (2014). URL https://www.nature.com/articles/nphoton.2014.135.
6. Posner, M. T. *et al.* High-birefringence direct UV-written waveguides for use as heralded single-photon sources at telecommunication wavelengths. *Optics Express* **26**, 24678–24686 (2018). URL https://www.osapublishing.org/oe/abstract.cfm?uri=oe-26-19-24678. Publisher: Optical Society of America.
7. Taballione, C. *et al.* A universal fully reconfigurable 12-mode quantum photonic processor. *Materials for Quantum Technology* **1**, 035002 (2021). URL https://doi.org/10.1088/2633-4356/ac168c. Publisher: IOP Publishing.
8. Taballione, C. *et al.* 8Ã—8 reconfigurable quantum photonic processor based on silicon nitride waveguides. *Optics Express* **27**, 26842–26857 (2019). URL https://www.osapublishing.org/oe/abstract.cfm?uri=oe-27-19-26842. Publisher: Optical Society of America.
9. Moss, D. J., Morandotti, R., Gaeta, A. L. & Lipson, M. New CMOS-compatible platforms based on silicon nitride and Hydex for nonlinear optics. *Nature Photonics* **7**, 597–607 (2013). URL https:

//www.nature.com/articles/nphoton.2013.183.

10. Ramelow, S. *et al.* Silicon-Nitride Platform for Narrowband Entangled Photon Generation. *arXiv:1508.04358 [physics, physics:quant-ph]* (2015). URL http://arxiv.org/abs/1508.04358. ArXiv: 1508.04358.

11. Arrazola, J. M. *et al.* Quantum circuits with many photons on a programmable nanophotonic chip. *Nature* **591**, 54–60 (2021). URL https://www.nature.com/articles/s41586-021-03202-1.

12. Guo, X. *et al.* Parametric down-conversion photon-pair source on a nanophotonic chip. *Light: Science & Applications* **6**, e16249–e16249 (2017). URL https://www.nature.com/articles/lsa2016249.

13. Ma, C. *et al.* Silicon photonic entangled photon-pair and heralded single photon generation with CAR > 12,000 and $g^{(2)}(0)$ < 0.006. *Optics Express* **25**, 32995–33006 (2017). URL https://www.osapublishing.org/oe/abstract.cfm?uri=oe-25-26-32995. Publisher: Optical Society of America.

14. Steiner, T. J. *et al.* Ultrabright entangled-photon-pair generation from an al ga as -on-insulator microring resonator. *PRX Quantum* **2**, 010337 (2021). URL https://link.aps.org/doi/10.1103/PRXQuantum.2.010337.

15. Luo, K.-H. *et al.* Direct generation of genuine single-longitudinal-mode narrowband photon pairs. *New Journal of Physics* **17**, 073039 (2015). URL https://doi.org/10.1088/1367-2630/17/7/073039. Publisher: IOP Publishing.

16. Luo, K.-H. *et al.* Nonlinear integrated quantum electro-optic circuits. *Science Advances* **5**, eaat1451 (2019). URL https://www.science.org/doi/10.1126/sciadv.aat1451. Publisher: American Association for the Advancement of Science.

17. Zhu, D. *et al.* Spectral control of nonclassical light using an integrated thin-film lithium niobate modulator. *arXiv* (2021). URL https://arxiv.org/abs/2112.09961.

18. Yu, M. *et al.* Femtosecond pulse generation via an integrated electro-optic time lens. *arXiv* (2021). URL https://arxiv.org/abs/2112.09204.

19. Davanco, M. *et al.* Heterogeneous integration for on-chip quantum photonic circuits with single quantum dot devices. *Nature Communications* **8**, 889 (2017). URL https://doi.org/10.1038/s41467-017-00987-6.

20. Zadeh, I. E. *et al.* Deterministic integration of single photon sources in silicon based photonic circuits. *Nano Letters* **16**, 2289–2294 (2016). URL https://doi.org/10.1021/acs.nanolett.5b04709.

https://doi.org/10.1021/acs.nanolett.5b04709.

21. Bacher, G. *et al.* Biexciton versus Exciton Lifetime in a Single Semiconductor Quantum Dot. *Physical Review Letters* **83**, 4417–4420 (1999). URL https://link.aps.org/doi/10.1103/PhysRevLett.83.4417. Publisher: American Physical Society.

22. Moreau, E. *et al.* Quantum Cascade of Photons in Semiconductor Quantum Dots. *Physical Review Letters* **87**, 183601 (2001). URL https://link.aps.org/doi/10.1103/PhysRevLett.87.183601. Publisher: American Physical Society.

23. Kiraz, A. *et al.* Photon correlation spectroscopy of a single quantum dot. *Physical Review B* **65**, 161303 (2002). URL https://link.aps.org/doi/10.1103/PhysRevB.65.161303. Publisher: American Physical Society.

24. Sun, X., Liu, H.-C. & Yariv, A. Adiabaticity criterion and the shortest adiabatic mode transformer in a coupled-waveguide system. *Opt. Lett., OL* **34**, 280–282 (2010). URL https://www.osapublishing.org/abstract.cfm?uri=ol-34-3-280.

25. Melo, E. G., Eshbaugh, W., Flagg, E. B. & Davanco, M. Multi-objective inverse design of solid-state quantum emitter single-photon sources (2022). URL https://arxiv.org/abs/2206.01043.

26. Ulhaq, A. *et al.* Detuning-dependent mollow triplet of a coherently-driven single quantum dot. *Optics Express* **21**, 4382–4395 (2013). URL http://www.opticsexpress.org/abstract.cfm?URI=oe-21-4-4382.

27. Konthasinghe, K. *et al.* Coherent versus incoherent light scattering from a quantum dot. *Physical Review B* **85**, 235315 (2012). URL https://link.aps.org/doi/10.1103/PhysRevB.85.235315. Publisher: American Physical Society.

28. Nguyen, H. S. *et al.* Optically gated resonant emission of single quantum dots. *Physical Review Letters* **108**, 057401 (2012).

29. Proux, R. *et al.* Measuring the Photon Coalescence Time Window in the Continuous-Wave Regime for Resonantly Driven Semiconductor Quantum Dots. *Physical Review Letters* **114**, 067401 (2015). URL https://link.aps.org/doi/10.1103/PhysRevLett.114.067401.

30. Muller, A. *et al.* Resonance fluorescence from a coherently driven semiconductor quantum dot in a cavity. *Physical Review Letters* **99**, 187402 (2007). URL https://link.aps.org/doi/10.1103/PhysRevLett.99.187402.
